# Implementing the Countrywide Mortality Surveillance in Action in Mozambique: How Much Did It Cost?

**DOI:** 10.4269/ajtmh.22-0438

**Published:** 2023-04-10

**Authors:** Safia S. Jiwani, Victor Américo Mavie, Emma Williams, Almamy Malick Kante, Agbessi Amouzou

**Affiliations:** 1 Department of International Health, Johns Hopkins Bloomberg School of Public Health, Baltimore, Maryland;; 2 National Institute of Health (INS), Maputo, Mozambique

## Abstract

Complete sample registration systems are almost inexistent in sub-Saharan Africa. The Countrywide Mortality Surveillance in Action (COMSA) project in Mozambique, a national mortality and cause of death surveillance system, was launched in January 2017, began data collection in March 2018, and covers over 800,000 population. The objectives of this analysis are to quantify the costs of establishing and maintaining the project between 2017 and 2020 and to assess the cost per output of the surveillance system using data from financial reports produced by the National Institute of Health in Mozambique. The program cost analysis consists of start-up (fixed) costs and average annual operating costs covering the period of maximum implementation in 700 clusters. The cost per output analysis quantifies the annual operating cost of surveillance outputs during the same period. Approximately two million dollars were spent on setting up the system, with infrastructure, technological investments, and training making up over 80% of these start-up costs. The average annual operating costs of maintaining COMSA was $984,771 per year, of which 66% were spent on wages and data collection incentives. The cost per output analysis indicates costs of $37–$42 per vital event captured in the surveillance system (deaths, pregnancies, pregnancy outcomes), $303–$340 per verbal and social autopsy conducted on a reported death, and a per capita cost of $1–$1.3. In conclusion, establishing COMSA required large costs associated with infrastructure and technological investments. However, the system offers long-term benefits for real-time data generation and informing government decision-making for health.

## INTRODUCTION

Data sources on vital statistics are essential for monitoring trends in population and health and for informing decision-making.^1^ These data can be obtained through national civil registration and vital statistics systems (CRVSs), which are sample registration systems (SRSs) that rely on a national random sample of communities, demographic surveillance systems, population surveys, and censuses.^2^ Data from population surveys and censuses are cross-sectional and therefore do not allow continuous monitoring or the production of real-time estimates; in contrast, although demographic surveillance systems are longitudinal, they are typically conducted at a small scale and do not produce national-level estimates. Therefore, CRVSs and sample registration systems are the most advantageous in terms of producing timely and continuous data on vital events at national and subnational levels. Over the past 30 years there has been little progress toward universal coverage of CRVSs in low- and middle-income countries (LMICs),^3^ and in sub-Saharan Africa in particular,^1^^,^^4^ where fewer than 10 countries have a complete death registration system.^4^ Complete death registration involves capturing the death event as well as the cause of death; however, with the vast majority of deaths occurring at home, causes of death often remain unknown.^5^

Several factors hinder the establishment of such national systems, including demand-side issues (e.g., weak political will and appreciation for the data generated, the lack of perceived benefit to registering vital events, the lack of social practice) and supply-side barriers (e.g., the inaccessibility of registration centers and services and financial constraints).^1^^,^^4^ Sample registration systems that produce accurate, continuous, and complete data on vital events and registration at national levels can be costly to establish and to maintain, and the cost is dependent on a variety of factors, including the size of the system, the of population it covers, and the frequency of contacts with the population.^6^ CRVSs are more costly and exhaustive in that they cover the entire country population rather than a random sample.^7^ The cost of building and maintaining CRVSs vary widely across settings and are not well documented in LMICs. A case study published in 2021 suggested that in Ghana, it cost on average $352,106 annually to set up and maintain the CRVS over the course of 10 years, with personnel costs accounting for over 70% of these costs.^8^ In contrast, Chile projected an annual cost of $45 million to maintain its CRVS in 2000.^1^ Recent advancements in digitization of CRVSs offer the potential for enhanced efficiency and reduced cost in the long run, although the initial stages may be cost intensive.^9^

The Countrywide Mortality Surveillance in Action (COMSA) project is a national mortality and causes of death surveillance system that was launched in Mozambique in January 2017. The objectives of this analysis are to quantify the program cost of establishing and maintaining the implementation of COMSA in Mozambique between 2017 and 2020 and to assess the cost per output of the system. Cost per output analyses aim to ascertain the annual operating costs associated with each of the outputs produced by the COMSA surveillance system with verbal and social autopsies.

## MATERIALS AND METHODS

In January of 2017, the government of Mozambique, in collaboration with Johns Hopkins University, received funding from the Bill & Melinda Gates Foundation to implement a national sample registration system called the COMSA, with verbal and social autopsy (VASA) to ascertain causes of death. The project was led by the Insituto Nacional de Estatisticas (INE) and the Insituto Nacional de Saúde (INS). COMSA covers 700 geographic clusters randomly selected across the 11 provinces in Mozambique, with each cluster containing approximately 300 households. The surveillance system was implemented in a phased manner, beginning data collection in five provinces in March 2018 and expanding to the remaining six provinces in October 2018. In each of the 700 clusters, a residing community member was recruited to serve as the community surveillance agent (CSA) for the surveillance system and electronically reported all pregnancies, pregnancy outcomes, and deaths that occurred within the assigned cluster, using a smartphone. For each death reported in the cluster, a trained VASA data collector followed up with the household and conducted a VASA interview using a tablet computer. COMSA recruited and trained 700 CSAs and more than 50 VASA data collectors, serving as the backbone of the surveillance system on the ground. Supervision is conducted using a cascade approach, whereby central level focal points based in Maputo at INS and INE are responsible for overseeing all project activities in their allocated provinces and liaise with provincial coordinators who maintain constant communication with VASA data collectors and CSAs. Details on the sampling framework and implementation are described elsewhere in this journal supplement.

Cost data were obtained from quarterly and annual financial reports produced by the INS in Mozambique, the institution responsible for the administrative and financial management of the project. We used the conceptual framework developed by Sohn et al.^10^ for assigning costs across three phases of program implementation, whose respective length may vary across settings: design, initiation, and maintenance. The design phase reflects preparation activities for launching the program, the initiation phase refers the costs of setting up the program, and the maintenance phase reflects costs to implement and maintain the program. Overall program costs were split into two types of costs: start-up (fixed) costs and operating (recurrent) costs.^11^^,^^12^ We further distinguished all costs between central and cluster levels within each phase.

The start-up costs were incurred during the design and initiation phases and reflect expenditures that were made for setting up the surveillance system during the first 2 years of the project. These costs were categorized into six main domains: infrastructure (i.e., vehicles), information technology (smartphones, tablets, laptops, etc.), field materials, training (of implementing institutions, CSAs, and VASA data collectors), baseline population and cluster mapping, and formative research. The operating costs were incurred during the maintenance phase and consisted of recurring expenditures related to the project’s implementation and maintenance. They were categorized into personnel costs (which included both central level salaries as well as provincial level salaries, incentives, and per diem payments for data collection), infrastructure maintenance, supervision and travel, communication, refresher trainings, and dissemination events. Average annual operating costs were calculated as the average annual expenditures of calendar years 2019 and 2020, during which the program was implemented at maximum scale in 700 clusters across 11 provinces in Mozambique.

In addition to start-up and operating costs, we presented separately the costs of the assessment activity, an endline evaluation conducted in all clusters between 2019 and 2020 by an external team of data collectors. The objective of the assessment was to validate the surveillance of vital events conducted by CSAs. We also presented operating costs disaggregated by province.

Our analysis includes project-related expenditures incurred in Mozambique, as well as some specific information technology (IT) purchases and software subscriptions made in the United States. Purchases from the United States were made in US dollars, whereas those from Mozambique were generally made in Mozambican metical and converted into US dollars at the exchange rate of the purchase date. Similarly, we restricted this analysis to in-country expenditures relating to the surveillance of vital events and VASA to reflect the program implementation costs in Mozambique, and we did not account for costs pertaining to technical assistance provided by the Johns Hopkins University. We also did not include additional special activities that were conducted in parallel to the national community surveillance, such as a CRVS pilot in the Inhambane province and a hospital-based surveillance at the central hospital in the Zambezia province.

The cost per output analysis aimed to quantify the annual operating cost of COMSA surveillance outputs. We estimated the annual operating cost per event reported, per VASA conducted, per cluster of 300 households covered, and per capita (based on the population covered in the 700 clusters). For this analysis, we used COMSA annual operating costs in 2019 and 2020, reflecting nationwide project implementation in 700 clusters during that period. We did not separate out costs by type of activity (surveillance versus VASA) because these activities are interlinked and we were unable to tease the costs apart. Events reported by the CSAs are needed for the VASA data collectors to follow-up and conduct a VASA interview; similarly, VASA data collectors contribute to CSA supervision; therefore, the system functions as a cascade. We included the unweighted cumulative number of pregnancies, pregnancy outcomes, and deaths reported between January 1, 2019, and December 31, 2020, and VASA interviews followed-up on deaths in that period. We divided the annual operating cost by the total number of events in each year to determine the annual operating cost per output.

## RESULTS

### Design and initiation phases: start-up costs.

The design phase of COMSA consisted of two main activities: 1) a formative research study conducted at the central level by a consultant at the beginning of the program to evaluate the role of key stakeholders and community leaders and to inform COMSA’s implementation and 2) a cartography activity conducted at the cluster level for household listing and delineation of cluster boundaries. These activities accounted for 14% of all start-up fixed costs, the remaining of which were incurred during the initiation phase of setting up the surveillance system.

Throughout the project spanning from 2017 to 2020, the implementation of COMSA in Mozambique cost $5,153,325, of which a little over $2,000,000 were spent on start-up investments in infrastructure, supplies, and training. Approximately half of this amount of fixed costs consisted of infrastructure and technology purchases needed for program implementation and data collection: vehicles, tablets, smartphones, laptops, as well as office IT equipment for each of the 11 provincial offices (desktops, printers, transformers). In addition, solar chargers were purchased in response to challenges reported on access to electricity to charge smartphones and tablets. The other half of the fixed costs included initial training of trainers and training of interviewers for the surveillance and VASA activities (32%) as well as the purchase of field materials (1.8%) (e.g., COMSA branded T-shirts and hats, backpacks for data collection teams, heavy duty stickers for household identification, and vehicle branding materials), and the formative research study (1.5%) and the cartography activity (12.4%), which were conducted during the design phase (Table 1 and Supplemental Figure 1). All technology supplies (tablets, smartphones, laptops, desktops, printers, transformers, and solar chargers) as well as heavy duty stickers and backpacks were purchased in the United States.

**Table 1:** Design and initiation phases: start-up fixed COMSA costs at central and cluster levels

Category	Description	Cost (US$)	Percentage
Design phase			
Central-level costs			
Formative research	Formative research study	29,400.0	1.5
Cluster-level costs			
Baseline population and cluster mapping	Household listing and delineating cluster boundaries, data collection and training materials	250,078.7	12.4
Initiation phase			
Central-level costs			
Infrastructure	Vehicles	800,000.0	39.5
Training	Training of trainers for CSA surveillance and VASA	143,245.0	7.1
Technology	Smartphones, tablets, laptops, desktops, monitors, printers, transformers, solar chargers, statistical software, international shipping	259,308.8	12.8
Cluster-level costs			
Field materials	T-shirts, hats, backpacks, household labels, banners, etc.	37,179.7	1.8
Training	Training of interviewers for CSA surveillance and VASA (travels, lodging, per diems, etc.)	503,787.8	24.9
Total		2,023,000.0	100.0

COMSA = Countrywide Mortality Surveillance in Action; CSA = community surveillance agent; VASA = verbal and social autopsy.

### Maintenance phase: operating costs.

The maintenance phase of the program consisted of operating costs to implement and maintain COMSA at the national level. We presented average annual operating costs during the two complete years of the project’s nationwide implementation in 700 clusters in 2019 and 2020 (Table 2 and Supplemental Figure 2). On average, $975,238 were spent annually: 66% of these costs were spent on personnel; this category includes both cluster-level personnel costs (salaries, incentives, and per diems for data collection) as well as central level in-country personnel costs (focal points, administrative/finance staff, and advisers). Infrastructure operating costs (21%) were also a major source of spending: vehicle maintenance and fuel were constant, costly challenges, costing approximately $36,000 and $90,000 annually, respectively, or on average $2,400 and $6000 per vehicle, although vehicles operating in large, rural, and hard-to-reach clusters required more maintenance and fuel compared with vehicles operating closer to urban areas. This category also included the cost of maintaining cloud servers, printing data collection forms and maps when needed, and unplanned emergency costs for personal protective equipment and biosafety during the COVID-19 pandemic; for instance, in 2020 COVID-related infrastructure purchases cost approximately $100,000. Communication costs contributed almost 7% of average annual operating costs and included central level communication ($30 per person monthly) and internet as well as all cluster-level staff purchases of cellphone data vouchers (including $5 and $10 per person monthly for CSA and VASA data collectors, respectively). Supervision by central-level staff included per diem payments and travel reimbursements, contributing to approximately 3% of operating costs. This entailed air or road travel costs for central-level staff based in Maputo to the provinces as well as a daily per diem rate of $100 per day during supervision to cover lodging and miscellaneous expenses. Supervision to each province was carried out on a bimonthly basis. Refresher trainings accounted for 3% of average annual operating costs: these were designed to retrain CSA and VASA interviewers on adequate data collection practices and methods as well as updates in data collection tools; they also provided an opportunity to train new data collectors given the high level of CSA turnover experienced during the project, documented elsewhere. Lastly, dissemination costs contributed to less than 1% annually, which included stakeholder meetings, national advisory board meetings, and capacity-building workshops.

**Table 2:** Maintenance phase: average annual operating costs at central and cluster levels (2019–2020)

Category	Description	Cost (US$)	Percentage
Central-level costs			
Personnel and incentives	Wages (INS, INE staff)	196,905.0	20.0
Infrastructure	Vehicle maintenance, fuel, cloud servers, printing, emergency infrastructure, etc.	202,766.9	20.6
Administration and logistics	Banking fees, tender announcements	10,025.1	1.0
Field supervision	Supervision of data collection and travels	26,839.6	2.7
Communication	Telephone, Internet	20,626.1	2.1
Dissemination	Stakeholder meetings, conferences, dissemination workshops, etc.	1,322.4	0.1
Cluster-level costs			
Personnel and incentives	Wages (Delegados, administrative/finance staff, coordinators, supervisors, VASA data collectors, CSA, drivers), incentives, health insurance, data collection per diems)	452,770.2	46.0
Communication	Staff communication plans	46,767.4	4.7
Refresher trainings	CSA and VASA refresher trainings	26,747.2	2.7
Total		$984,771.0	100.0

CSA = community surveillance agent; INE = Insituto Nacional de Estatisticas; INS = Insituto Nacional de Saúde; VASA = verbal and social autopsy.

Average annual operating costs were further broken down and presented for each of the 11 provinces where COMSA was implemented. Supplemental Table 1 reflects costs incurred at the provincial level and shows that Manica, Cabo Delgado, Tete, and Zambezia incurred the highest operating costs at provincial levels compared with other provinces; these represent the provinces in which COMSA had a larger number of clusters and field teams posted. However, at the cluster level, Sofala, Gaza, Niassa, and Nampula incurred the highest average cost per cluster. In general, personnel and supervision accounted for the largest cost buckets across all provinces. Infrastructure maintenance and refresher training costs had large variability across provinces because they were implemented on an “as-needed” basis. Note that central-level personnel and supervision expenses were not included in this table because they were not incurred at provincial levels.

### Operating cost per output.

COMSA outputs consist of reported events by CSAs (pregnancy outcomes, pregnancies, and deaths), VASAs conducted, clusters, and populations covered by the project. Table 3 indicates that 29,237 and 20,776 events were reported by the CSAs in 2019 and 2020, respectively; 3,627 and 2,571 respective VASAs were conducted on deaths followed up by VASA interviewers; and 700 clusters were included in COMSA, which covered a population of 865,486 individuals, based on the surveillance data extracted in May 2021. It is important to note that COMSA outputs in 2020 were severely affected by the COVID-19 pandemic: from March to July 2020, surveillance activities in all 700 clusters were halted, which explains the sharp decline of approximately 30% in the number of events reported in 2020 compared with 2019 (Table 3).

**Table 3:** COMSA outputs in 2019 and 2020

	2019	2020
Pregnancies reported	10,778	7,631
Deaths reported	3,986	2,653
Pregnancy outcomes reported	14,473	10,492
All events (pregnancy, pregnancy outcome, death)*	29,237	20,776
VASA conducted	3,627	2,571
Clusters	700	700
Population*	865,486	865,486

COMSA = Countrywide Mortality Surveillance in Action; VASA = verbal and social autopsy.

* Number of events and population are based on surveillance data extracted on May 28, 2021.

Each event reported by CSAs cost $38 and $42 annually in 2019 and 2020, respectively, whereas each VASA conducted for deaths followed up by a VASA interviewer cost $303 and $339, respectively. Regarding the magnitude of the project coverage in 2019 and 2020, it cost $1,568 and $1,245 per cluster of approximately 300 households covered, respectively, and $1 per capita based on the population covered in 700 clusters to implement the project annually (Table 4).

**Table 4:** Annual operating cost (in US$) per output in 2019 and 2020

Year	Annual operating cost	Annual cost per event	Annual cost per VASA	Annual cost per cluster	Annual cost per capita
2019	1,097,847.1	37.6	302.7	1,568.4	1.3
2020	871,693.0	42.0	339.1	1,245.3	1.0

VASA = verbal and social autopsy.

### 2020 Assessment: endline surveillance evaluation.

The endline surveillance evaluation conducted between 2019 and 2020 incurred a total expenditure of $650,843.6 in the 700 COMSA clusters. Table 5 indicates that 67% of this cost was spent on personnel wages and incentives and 24% on infrastructure (travels, vehicles, fuel, etc.). The remaining 9% was spent on training of data collectors, communications, printing, and other miscellaneous items.

**Table 5:** Endline surveillance evaluation costs (2019–2020)

Category	Description	Cost (US$)	Percentage
Personnel and incentives	Provincial and central team wages, supervisors per diems, data collectors per diems, guide fees	436,231.2	67.0
Infrastructure and travels	Travel tickets, vehicle rental, fuel, maintenance	156,905.4	24.1
Training	Trainings and refresher trainings	7,918.9	1.2
Other	Catering, printing, communication	49,788.0	7.6
Total		650,843.6	100.0

## DISCUSSION

Our analysis suggests that setting up and maintaining a national mortality surveillance system in 700 clusters throughout Mozambique cost $5,804,169 over the course of 4 years from 2017 to 2020, including the 2020 assessment. This covers reporting of vital events through the community surveillance as well as ascertainment of cause of death through VASAs carried out for all reported deaths. Importantly, 35% of these costs consisted of the project start-up investments in infrastructure, technology, and materials for data collection; initial training of trainers and interviewers; and costs of conducting a formative research study and cartography activities. Given the higher costs and administrative challenges we encountered with purchasing items such as laptops, smartphones, and tablets through the government institutions in Mozambique, we decided to purchase them in the United States and ship them overseas; this has been done in other projects^13^ and significantly reduced the cost (which could have likely doubled otherwise) as well as the time it took to set up the project. Replacement of cell phones lost due to theft or damage was a common challenge across all provinces in Mozambique, and this was associated with increased infrastructure cost, which has also been reported in another study relying on electronic data capture in rural settings.^14^ In the case of COMSA, all 700 cellphones initially purchased needed to be replaced due to damage or theft, costing an additional $81,000 to the project. In contrast, the benefits of electronic data collection have been reported in several studies,^13^^,^^15^^,^^16^ including a demographic surveillance in rural Malawi where focus groups revealed that it was more cost effective than paper-based data collection because devices can be used across several projects and serve multiple purposes.^13^ Importantly, electronic data collection provides significant improvements in the timeliness of reporting and in data quality.^9^ Additionally, a study on the use of smartphones for verbal autopsy in rural China suggested easier data collection reported by interviewers.^16^

Fixed costs aside, implementing and maintaining COMSA cost on average $984,771 per year (using data from 2019 to 2020), which covered personnel costs, infrastructure maintenance, supervision of fieldwork activities, refresher trainings, communication, dissemination, and administrative costs. Other studies assessing national platforms with vital event reporting have also highlighted the large costs associated with maintaining such programs and the challenges around resource allocation.^1^ An economic analysis compared the costs of a CRVS with that of the census and a population-based survey in Lao People’s Democratic Republic over a 20-year period and showed that the CRVS ranked best in terms of cost-effectiveness, suggesting that investing in a CRVS would prove to be more economical than relying on censuses or surveys for vital statistics.^2^

In the COMSA context, personnel costs were the largest category because they also included salary and incentives for data collection activities. In addition, maintenance costs and fuel were unexpectedly high every year: maintenance of vehicles and fuel proved to be extremely costly in Mozambique and indispensable to navigate poor road infrastructure and heavy rainy seasons; these costs exceeded our budgeted estimates, and we had to adapt accordingly. These high operational costs related to topography, transport, and communications have also been documented in another sentinel sample registration system in Tanzania.^6^ In addition to these, emergency post hoc costs were included in our analysis: specifically, in 2020 the project incurred unplanned expenditures to comply with restrictions and safety protocols during the COVID-19 pandemic. Data collection activities were halted between March and July 2020 upon a mandated suspension of all field activities by the National Institute of Health (INS), which led to a lower number of events reported and VASAs conducted in 2020 compared with 2019. In accordance with INS protocols, we held a biosafety training and provided personal protective equipment to all fieldworkers and staff to ensure compliance with safety measures. These activities led to an approximately $100,000 increased emergency expenditure in 2020.

Our operating cost per output analysis suggested that each event reported cost between $38 and $42 in 2019 and 2020, and each VASA conducted on reported deaths cost between $303 and $330 during the same period. The COMSA per-capita operating annual cost was $1. Studies assessing cost-per output of national mortality surveillance systems are scarce; Somda et al.^17^ reported average costs of integrated disease surveillance and response systems ranging from $0.02 per capita in Mali to $0.16 in Eritrea between 2002 and 2005. In India, a cost analysis of a verbal autopsy–based mortality surveillance covering a population of around 185,000 in Andhra Pradesh suggested an annual cost of $17 per death for the first year, followed by $12 for subsequent years, while the annual cost of maintaining the surveillance system was $19,800.^18^ These costs appear to be lower than what we have reported for COMSA in Mozambique, and it has been shown that factors such as the size of the system, number of clusters covered, and frequency of contact with households heavily influence program cost.^6^ As a comparison, the COMSA system covered a population of over 800,000 individuals across all 11 provinces in Mozambique.

Our study has several strengths. To the best of our knowledge, it is the first cost assessment of a national mortality surveillance system in the sub-Saharan African context,^4^ and we hope that our findings can inform the implementation and planning of similar projects in the region. We were also able to distinguish fixed project costs from recurrent operating costs; this was a crucial aspect of our analysis and highlighted the heavy start-up costs of establishing the COMSA system in Mozambique. Our study also has important limitations: First, we were not able to tease out surveillance activity costs conducted by the CSAs from verbal autopsy activities led by the VASA interviewers. Although event reporting and VASA interviews were implemented by different cadres of data collectors, the COMSA system was built using a cascade model whereby reporting of deaths by the CSAs were a pre-requisite to VASAs conducted on these reported deaths; similarly, field supervision activities and data collection costs were not reported separately between surveillance and verbal autopsy activities. We believe that presenting distinct surveillance and verbal autopsy costs would be an inadequate representation of the COMSA system as a whole, given that the project operated in a way that integrated both sets of activities closely and surveillance costs were needed for VASA implementation. Since COMSA data collection activities were suspended between March and July 2020, the number of events used as a denominator for the cost per output analysis were likely under-reported in 2020. Similarly, relative to previous years, there was also less spending in 2020 in terms of travel, supervision and training, given restrictions during the pandemic. We did not include costs associated with other COMSA activities conducted at a smaller-scale in specific provinces, such as a CRVS pilot in Inhambane, a hospital-based surveillance in one hospital and minimally invasive tissue sampling in Zambezia; we considered these components of the program to be distinct from the main community surveillance and therefore were beyond the scope of this analysis. We attempted to distinguish costs incurred at central and cluster-levels, but for some activities the costs may have been shared. And finally, the costs included in this analysis are specific to the Mozambican economic and trade context (i.e., wages, price of fuel, vehicle maintenance, technology equipment, etc.); these are likely to vary significantly across countries within the sub-Saharan African region.

In the context of the COVID-19 pandemic, COMSA has proven to be a valuable resource for monitoring excess mortality due to COVID-19 because it is the only platform that provides data on community deaths in the country.^7^ Similarly, COMSA fieldworkers are being trained and mobilized to support the efforts of the government of Mozambique in case identification, referral, contact tracing, and health promotion in a highly evolving context of increased surveillance needs to manage the pandemic. Therefore, the value of COMSA extends beyond community surveillance, and its benefits are not fully reflected in our cost analysis.

In conclusion, our program cost analysis from the Mozambican COMSA experience suggests that establishing and maintaining a national surveillance system with community-based event reporting and VASA is resource intensive. However, it is important to keep in mind that a large proportion of the costs relate to laying the foundation of the system and investing in the technological equipment needed for it to function, which were incurred in the initiation phase of the program. Although securing such large amounts of funding for setting up similar national surveillance systems is challenging, these costs are expected to decrease over time, and it is essential to consider the long-term benefits that these systems can offer in terms of high-quality data generation and informing critical decision-making, particularly in the current contexts of the COVID-19 pandemic, as well as their potential superior cost-effectiveness in comparison to censuses and population-based surveys. Further research is needed to assess the sustainability of such resource-intensive systems in a context of fragile systems and limited resources and the ways in which COMSA-like systems could be maintained in a more affordable manner in the long-run while continuing to serve as a national platform for continuous and real-time generation of vital statistics.

## Financial Disclosure

Financial support: The COMSA Mozambique project is funded by the Bill & Melinda Gates Foundation (grant number OPP1163221).

## Supplemental Materials


Supplemental materials
