# Supplementary material for: Implementing the Countrywide Mortality Surveillance in Action in Mozambique: How Much Did It Cost?
Source: Am J Trop Med Hyg. 2023 Apr 10;108(5 Suppl):40–6. doi: 10.4269/ajtmh.22-0438 (PMC10160867; doi:10.4269/ajtmh.22-0438)
Supplement: Supplementary file 1 [file tpmd220438.SD1.pdf]

Supplementary Figure 1. Start-up fixed COMSA costs (%)

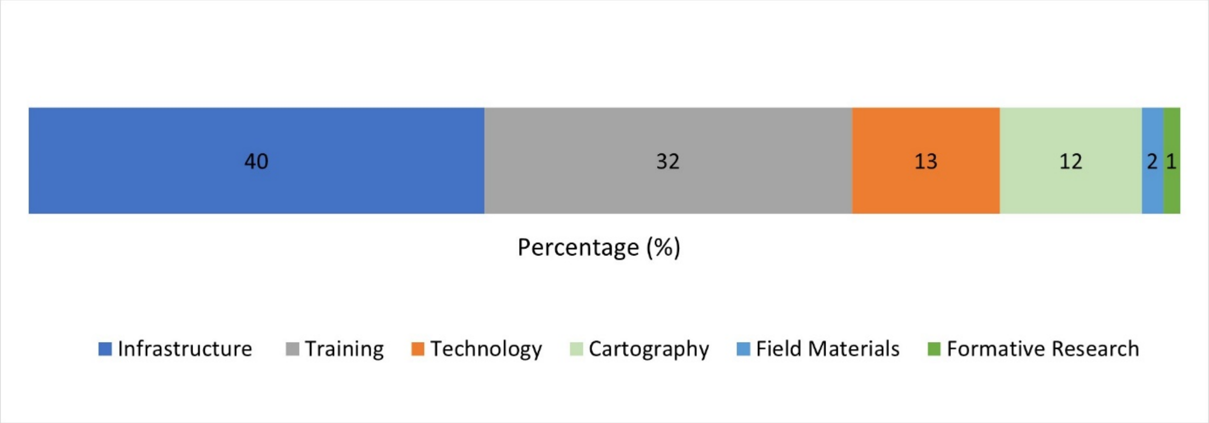

Supplementary Figure 2. Average annual COMSA operating costs (%)

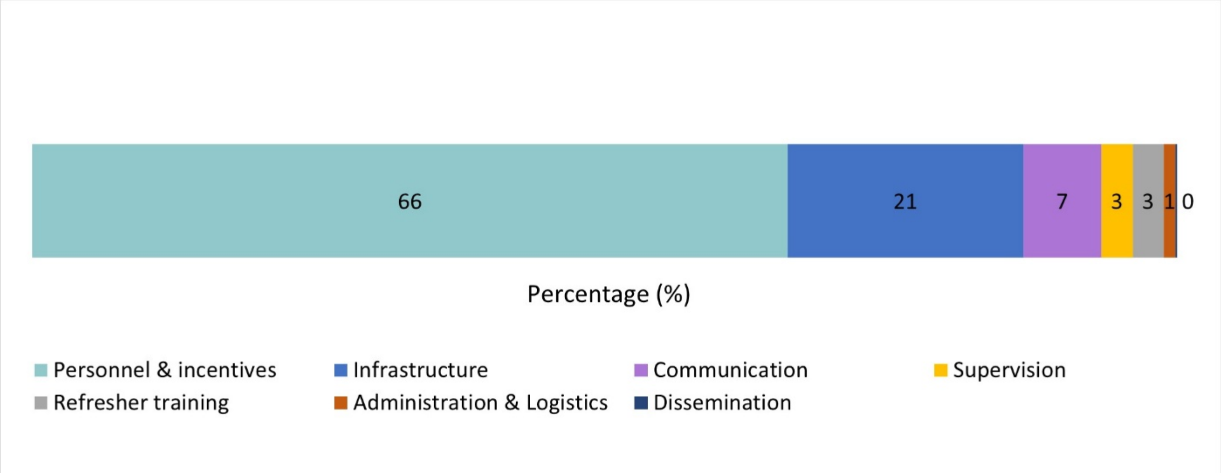

Supplementary Table 1. Average annual operating costs at provincial levels (2019-2020)

**Categories**	Manica n=85	Niassa n=40	Inhambane n=49	Gaza n=36	Maputo Provincia n=36	Maputo Cidade n=35	Cabo Delgado n=113	Tete n=106	Zambézia n=118	Nampula n=53	Sofala n=29
Personnel & incentives ($)	35,642.2	19,084.0	23,771.8	19,698.1	19,160.9	19,129.4	44,556.7	32,886.4	42,676.3	26,389.9	15,520.6
Infrastructure & maintenance ($)	14,250.0	10,180.4	7,417.5	10,876.6	5,524.9	3,435.9	19,689.1	6,227.4	8,103.1	11,817.1	5,161.4
Administration/logistics & other ($)	487.2	497.9	70.8	116.7	209.1	52.6	322.9	342.6	335.0	240.7	128.0
Supervision (provincial only) ($)	16,152.9	8,973.9	10,619.1	3,655.6	3,655.6	3,655.6	14,866.7	14,866.7	14,866.7	8,495.2	8,495.2
Communication ($)	5,985.9	1,692.1	3,313.6	2,699.8	3,284.5	2,191.5	6,957.4	4,129.0	5,429.4	3,943.9	1,378.0
Refresher training ($)	1,038.1	488.5	598.4	439.7	439.7	439.7	4,699.7	5,574.6	8,720.3	2,307.1	2,014.0
**Total average annual operating costs per province ($)**	**73,556.3**	**40,916.8**	**45,791.2**	**37,486.5**	**32,274.7**	**28,904.7**	**91,092.5**	**64,026.7**	**80,130.8**	**53,193.9**	**32,697.2**
**Total average annual operating costs per cluster per province ($)**	**865.4**	**1022.9**	**934.5**	**1041.3**	**896.5**	**825.8**	**806.1**	**604.0**	**679.1**	**1003.7**	**1127.5**

**n= number of COMSA clusters per province*

Supplementary Table 2. Assessment costs per province (2019-2020)

**Categories**	Manica<br>n=85	Niassa<br>n=40	Inhambane<br>n=49	Gaza<br>n=36	Maputo Provincia<br>n=36	Maputo Cidade<br>n=35	Cabo Delgado<br>n=113	Tete<br>n=106	Zambézia<br>n=118	Nampula<br>n=53	Sofala<br>n=29
Personnel & incentives ($)	52,970.9	24,927.5	30,536.2	22,434.7	22,391.8	21,858.9	70,419.3	66,052.9	73,536.1	33,028.9	18,072.4
Infrastructure, maintenance & travel ($)	19,052.8	8,966.0	10,983.4	8,069.4	8,073.5	7,841.0	25,329.0	23,760.0	26,449.8	11,880.0	6,500.4
Training ($)	961.6	452.5	554.3	407.3	407.3	396.8	1,278.3	1,199.2	1,334.9	599.6	328.1
Other (communication, catering, etc.)	6,045.7	2,845.0	3,485.2	2,560.5	2,560.5	2,489.3	8,037.2	7,539.3	8,392.8	3,769.7	2,062.6
**Total ($)**	**79,031.0**	**37,191.1**	**45,559.1**	**33,472.0**	**33,433.0**	**32,586.1**	**105,063.9**	**98,551.4**	**109,713.6**	**49,278.2**	**26,963.5**

**n= number of COMSA clusters per province*
